# Role of Adipose Tissue-Derived Autotaxin, Lysophosphatidate Signaling, and Inflammation in the Progression and Treatment of Breast Cancer

**DOI:** 10.3390/ijms21165938

**Published:** 2020-08-18

**Authors:** David N. Brindley, Xiaoyun Tang, Guanmin Meng, Matthew G. K. Benesch

**Affiliations:** 1 Department of Biochemistry, University of Alberta, Edmonton, AB T6G 2S2, Canada; xtang2@ualberta.ca (X.T.); guanmin@ualberta.ca (G.M.); benesch@ualberta.ca (M.G.K.B.); 2 Cancer Research Institute of Northern Alberta, University of Alberta, Edmonton, AB T6G 2S2, Canada; 3 Discipline of Surgery, Faculty of Medicine, Memorial University of Newfoundland, St. John’s, NL A1B 3V6, Canada

**Keywords:** adiponectin, chemokines, chemotherapy, cytokines, fibrosis, macrophages, radiotherapy, tumor microenvironment

## Abstract

Autotaxin (ATX) is a secreted enzyme that produces lysophosphatidate (LPA), which signals through six G-protein coupled receptors, promoting tumor growth, metastasis, and survival from chemotherapy and radiotherapy. Many cancer cells produce ATX, but breast cancer cells express little ATX. In breast tumors, ATX is produced by tumor-associated stroma. Breast tumors are also surrounded by adipose tissue, which is a major bodily source of ATX. In mice, a high-fat diet increases adipocyte ATX production. ATX production in obesity is also increased because of low-level inflammation in the expanded adipose tissue. This increased ATX secretion and consequent LPA signaling is associated with decreased adiponectin production, which results in adverse metabolic profiles and glucose homeostasis. Increased ATX production by inflamed adipose tissue may explain the obesity-breast cancer association. Breast tumors produce inflammatory mediators that stimulate ATX transcription in tumor-adjacent adipose tissue. This drives a feedforward inflammatory cycle since increased LPA signaling increases production of more inflammatory mediators and cyclooxygenase-2. Inhibiting ATX activity, which has implications in breast cancer adjuvant treatments, attenuates this cycle. Targeting ATX activity and LPA signaling may potentially increase chemotherapy and radiotherapy efficacy, and decrease radiation-induced fibrosis morbidity independently of breast cancer type because most ATX is not derived from breast cancer cells.

## 1. Autotaxin and LPA Metabolism

**Figure 1:**
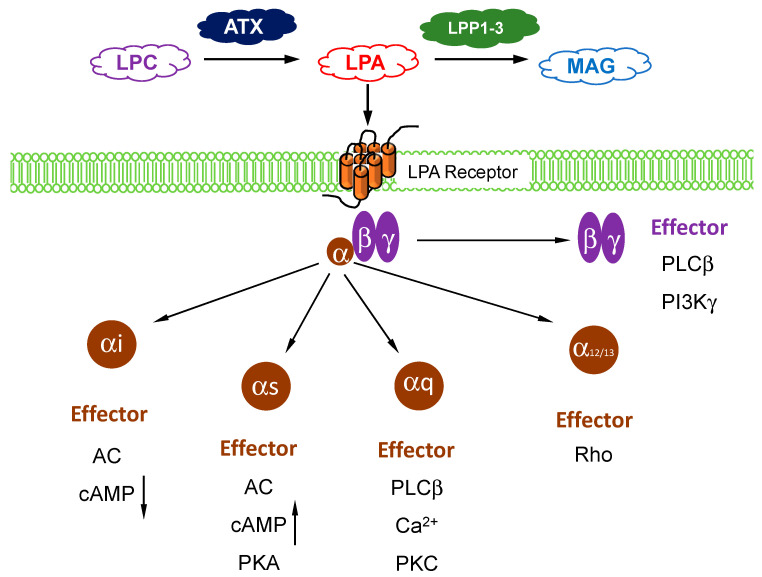
Overview of lysophosphatidate (LPA) signaling pathway. Extracellular LPA is produced from the enzymatic action of autotaxin (ATX) on lysophosphatidylcholine (LPC). LPA is degraded by lipid phosphate phosphatases (LPP)1–3 into inactive monoacylglycerol (MAG). LPA signals through at least six known G-protein coupled receptors (with three sub-units) to mediate its downstream cellular effects, which are dependents on the coupling and/or subunit type.

Autotaxin (ATX) belongs to a family of ectonucleotide pyrophosphatase/phosphodiesterases (ENPPs) and its gene name is *ENPP2* [1]. There are five other members of this family and these hydrolyze phosphodiester bonds in nucleotide phosphates [2]. By contrast, secreted ATX acts primarily as a lysophospholipase D, which converts extracellular lysophosphatidylcholine (LPC) into lysophosphatidate (LPA). The affinity of ATX for LPC is ~10-fold higher than for nucleotide substrates [3]. ATX was discovered in culture medium from melanoma cells because of its effects in stimulating cell migration [4]. It was not until a decade later that this cell migration effect was shown to depend on its production of lysophosphatidate (LPA) [5,6]. In fact, most of the biological functions of ATX are attributed to signaling by LPA [7]. ATX acts as the “gatekeeper” to control LPA signaling through a family of six G protein-coupled receptors (Figure 1). The LPA receptors are widely expressed in different cells and they regulate a wide range of signaling pathways through their coupling to Gi, Gs, Gq, and G12/13 (Figure 1) [8,9].

The Km of ATX for LPC is ~100 µM [10] whereas the concentrations of LPC in human blood are >200 µM [11]. LPA concentrations in plasma are normally about between 0.1–1 µM [10] and most of this LPA is generated through ATX (Figure 1). This is demonstrated in work with mice that were treated with ATX inhibitors or *ENPP2*^+/−^ mice that were used, since there were 50–90% decreases in plasma LPA levels [12,13,14,15]. ATX is essential for vasculogenesis and early tissue development in the embryo since *ENPP2*^−/−^ mice only survive until about embryonic day 9.5 when they die with vascular and neural tube defects [12,16].

The turnover of extracellular LPA is mainly controlled by the ecto-activities of a family of three lipid phosphate phosphatases (LPPs), LPP1, LPP2, and LPP3, which are encoded by three separate genes *PLPP1*, *PLPP2*, and *PLPP3*, respectively (Figure 1) [17,18,19,20]. The LPPs dephosphorylate a large variety of lipid phosphates and lipid pyrophosphates. They are integral membrane proteins, which are expressed by most cells, and they belong to a conserved phosphatase/phosphotransferase superfamily. The active sites of these enzymes are composed of three separate domains, which are expressed on the outer surface of cells where the LPPs are located on the plasma membranes [21]. This enables the LPPs to degrade extracellular LPA and its sphingolipid counterpart, sphingosine 1-phosphate (S1P), which signals through five G-protein coupled receptors. The turnover of extracellular LPA is very rapid with a t_1/2_ of about 1 min, which demonstrates the dynamic interaction of the activities of ATX and the LPPs.

It was proposed that LPA turnover is self-regulated by product inhibition since ATX activity was claimed to be inhibited by LPA or S1P [22]. This claim turned out to be a consequence of an artifact of the assay system used for ATX because of the low concentration of fluorescent ATX substrate that was employed [23]. Increasing the concentration of the fluorescent ATX substrate or using physiological concentrations of LPC overcame the inhibitions caused by LPA and S1P. However, S1P and LPA do exert a feedback on ATX secretion, but this is achieved by decreasing ATX expression at the transcription level [23]. Intravenously injected ATX [24] or LPA [25,26] are removed rapidly from the blood and this involves hepatic uptake.

The negative regulation of ATX transcription by LPA and S1P is overcome by the effects of inflammatory cytokines and chemokines, which increase ATX production and secretion [23]. This explains why there is increased expression of ATX together with high LPA concentrations in inflammatory diseases including cancers. This situation is enhanced in cancers where there is decreased expression of LPP1 and LPP3, which leads to slower turnover of LPA [21,27]. In addition, the low expression of LPP1 makes cancer cells hypersensitive to the effects of LPA. Significantly, decreasing the extent of the inflammatory milieu with dexamethasone attenuates LPA signaling by decreasing ATX secretion and the production of LPA1 receptors and by increasing the expression of LPP1 [28].

## 2. Role of ATX Activity in Wound Healing, Chronic Inflammation, and Cancer

One of the important functions of ATX and LPA signaling is in wound healing. ATX is secreted by activated platelets and secretion from tissues is increased by inflammation that is caused by tissue damage or infections [29,30]. The consequent increase in LPA signaling stimulates wound repair by activating the migration and division of fibroblasts and keratinocytes in the wounded area [31] and by facilitating collagen deposition [32], which leads to the formation of scar tissue. LPA also stimulates angiogenesis by activating the migration and proliferation of vascular endothelial cells [33,34] to provide the new blood vessels necessary for tissue repair. LPA also induces lymphocyte homing [35] and transformation of monocytes to macrophages [36], which is an important part of the host defense system. When the wound is healed, inflammation resolves and the secretion of ATX falls back to basal conditions [29,37].

There are many conditions in which inflammation is not resolved. These chronic inflammatory conditions include pulmonary fibrosis, cirrhosis, obesity, rheumatoid arthritis, inflammatory bowel disease, cardiovascular diseases, and cancers [8,38]. In fact, the wound healing functions of ATX and LPA are hijacked in cancers (wounds that do not heal) [39,40,41,42]. Inflammation and decreased acquired immune responses are “hallmarks” of cancer [8,41,43]. Chronic LPA signaling enables cancer cells to evade the immune system [44,45,46] and the activation of LPA5 receptors suppresses the function of CD8+ cytotoxic T cells by inhibiting the mobilization of intracellular Ca^2+^ and extracellular signal-related kinases (ERK) activation [47]. LPA when bound to low-density lipoproteins (LPL) promotes platelet activity, which leads to stress fiber production and vascular endothelial damage. Mechanistically, LPA signaling through TLR4 receptors, which is a toll-like receptor partly responsible for innate immune system activation, upregulates nuclear factor kappa-light-chain-enhancer of activated B cells (NFκB) activation. This may contribute to the creation of a chronic inflammatory milieu central to the progression of atherosclerosis [48,49]. LPA also increases vascular endothelial growth factor (VEGF) production, which stimulates the angiogenesis needed for tumor growth [50]. LPA signaling is generally increased in cancers because of the high secretion of ATX and the low expressions of LPP1 and LPP3 [51]. ATX concentrations are correlated with invasiveness [8,10,52] and the ATX gene (*ENPP2*) is among the 40–50 most up-regulated genes in metastatic tumors [53,54,55].

The imbalance in expression between macrophage types M1 and M2 is implicated in the development of auto-immunity and obesity in murine models [56]. Typically, M1 macrophages are labeled as classically activated or inflammatory macrophages and M2 macrophages as alternatively activated, or wound-healing. Normally, macrophages retain their imprinted phenotype but they have sufficient plasticity to be remodeled as needed in response to acute inflammatory cytokines, which increases the M1/M2 ratio [57]. Adiponectin contributes toward an anti-inflammatory profile with increased M1 macrophage expression in adiponectin knockout mice, and blocks M1 marker expression in human circulating monocyte-derived macrophages and stromal vascular fraction cells in human subcutaneous fat [58].

Recently, it was reported that tumor-associated macrophages may be the predominant source of LPA production in the ascites of ovarian cancer patients, and that CD163+CD206+ tumor-associated macrophages play an essential role as the main producers of ATX and phospholipase A2s (PLA2s) [59]. While tumor cells express predominantly LPA1–3 receptors, macrophages and T cells selectively express LPA5 and LPA6 receptors at high levels, which points to cell type-selective LPA signaling pathways in a cancer microenvironment [42,60]. Antagonizing the actions of the LPA5 receptor has been proposed as an essential target for the immunological control of cancer progression [60].

## 3. Role of Adipose Tissue as a Source of ATX Production in Breast Cancer

**Figure 2:**
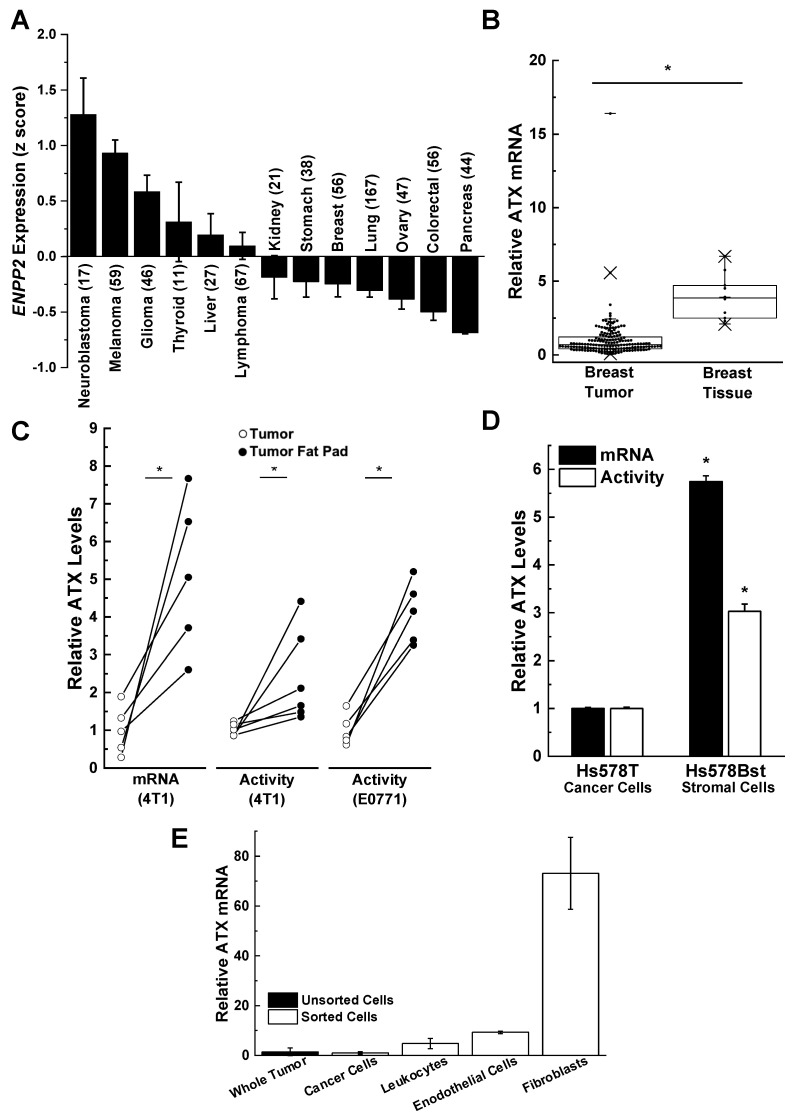
Breast cancer cells are poor producers of autotaxin (ATX) compared to adjacent adipose tissue tumor-associated fibroblasts. (**A**) Human breast cancer cells express little ATX compared to other neuroblastoma, melanoma, glioma, thyroid, and liver cancer cells. Results are means ± SEM. Numbers in parentheses indicates the number of cell lines. Results taken from cBioPortal (www.cbiportal.org) [63,64] and are reproduced from Reference [29] with permission. (**B**) ATX mRNA expression in 176 human breast tumors and 10 normal breast tissue specimens. Box plots show minimum, mean, and maximum values, 25th, 50th, and 75th percentiles (box), and 1st and 99th percentiles. Results are expressed relative to the mean of the breast tumor results, which were given the value of 1. * *p* < 0.001. Adapted from Reference [67]. (**C**) ATX, mRNA, and activity levels are significantly lower in tumors compared to adjacent fat pads in orthotopic syngeneic and immunocompetent mouse models (4T1/BALB/C, E0771/C57BL/6) * *p* < 0.05 by a paired *t*-test. Results are expressed relative to the mean of the breast tumor results, which were given the value of 1. Includes results adapted from Reference [14]. (**D**) Relative ATX mRNA and activity levels in patient-matched Hs578T breast cancer cells and Hs578Bst stromal cells. Results are means ± SEM from three independent experiments. * *p* < 0.05 vs. Hs578T breast cancer cells. Adapted from Reference [67]. (**E**) ATX expression in mouse 4T1 tumors comes predominantly from cancer-associated fibroblasts. Whole 4T1 tumors were enzymatically digested and sorted by flow cytometry for cancer cells (epithelial cells) using EPCAM (epithelial cell adhesion molecule), leukocytes using CD-45, endothelial cells using CD-31, and cancer-associated fibroblasts using platelet-derived growth factor alpha (PDGFα). ATX mRNA levels are expressed relative to those in the whole tumor. Results are means ± SEM from three independent experiments for whole tumor and cancer cells, and means ± range for two independent experiments for leukocytes, endothelial cells, and fibroblasts.

ATX is secreted directly in highly significant amounts by melanoma, glioblastoma, glioma, and thyroid tumors when compared to stomach, breast, lung, ovary, colorectal, and pancreatic tumors [61,62,63,64] (Figure 2A). ATX mRNA levels and ATX activity are relatively low in human breast tumors compared to adjacent breast tissue rich in adipose tissue (Figure 2B). A similarly low ATX activity is seen in mouse 4T1 and E0771 breast tumors compared to adjacent adipose tissue [7,62,65,66,67] (Figure 2C). This relationship is also illustrated by the comparison of human Hs578T breast cancer cells relative to Hs57Bst stromal cells isolated from the same tumor (Figure 2D). When the cells from mouse 4T1 breast tumors were separated after collagenase digestion, the majority of the ATX mRNA was associated with the fibroblast fraction rather than with endothelial cells or leukocytes. The breast cancer cells had very low ATX mRNA expression (Figure 2E). These experiments on ATX mRNA expression provide information on where ATX is produced before it is secreted into the tumor microenvironment where it attaches to adjacent cells including cancer cells by binding to integrins and syndecan-4. This binding appears to selectively channel LPA signaling to LPA receptors [68,69,70,71]. It is, therefore, concluded that most of the ATX that mediates LPA signaling is produced by adipocytes, fibroblasts, and tumor-associated stromal cells rather than the breast cancer cells themselves.

**Figure 3:**
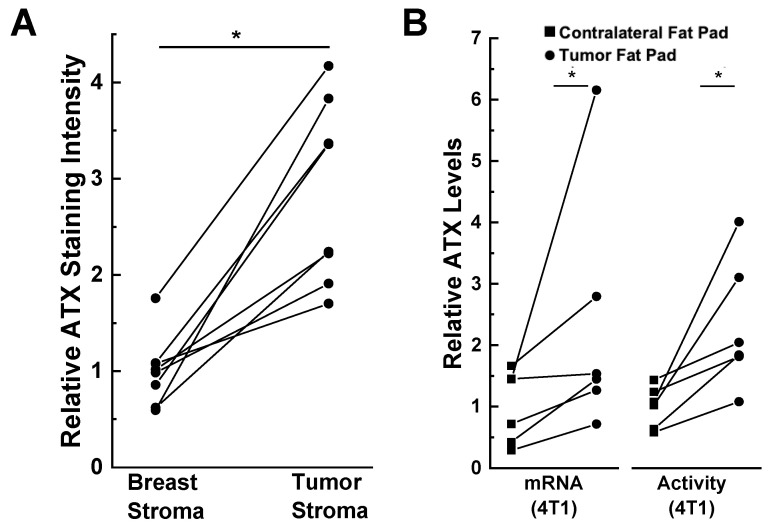
ATX is induced in tumor-associated compared to normal breast adipose tissue. (**A**) ATX immunohistochemical staining is increased in human tumor stroma compared to adjacent breast stroma. * *p* < 0.001 by paired *t*-test. Staining intensity was quantified by ImageJ [(NIH), Bethesda, MD, USA] and the results were adapted from Reference [67]. (**B**) ATX mRNA and activity levels are higher in tumor-bearing mammary fat pads compared to unaffected contralateral fat pads in a 4T1 orthotopic, synergistic, and immunocompetent BALB/C mouse model. ATX staining and mRNA levels are expressed relative to the breast stroma and contralateral fat pad, respectively. * *p* < 0.05 by paired *t*-test. Results adapted from Reference [14].

The presence of the tumor also influences this expression of ATX. This is illustrated by immunostaining of human tissues where ATX is present at higher concentrations in human breast tumor stroma compared to the adjacent breast stroma (Figure 3A) [67]. Furthermore, ATX mRNA expression and activity in the fat pad adjacent to 4T1 breast tumors in mice is higher than in the contralateral fat pad that did not contain a tumor (Figure 3B) [14]. Popnikolov et al. [72] also used immunostaining for ATX and showed positivity for ductal carcinomas. There was strong ATX staining in peritumoral fibroblasts, whereas the cancer cells were weakly positive. In addition, ATX staining was low in normal ducts and lobules compared to the carcinomas. It is, therefore, important to consider where ATX is produced and where the secreted protein is expressed. ATX production in breast cancer cells and normal epithelial cells is low compared to that in breast adipocytes and fibroblasts.

**Figure 4:**
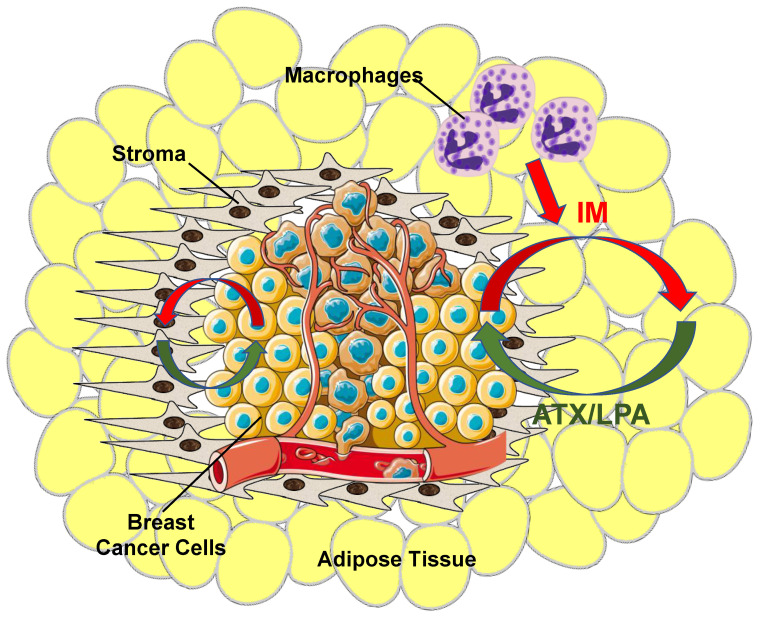
Overview of ATX/LPA signaling within the breast tumor microenvironment. Breast cancer cells produce virtually no ATX relative to tumor stroma and surrounding adipose tissue. Instead, as cancer cells grow, they establish an inflammatory milieu where inflammatory mediators (IM) (red arrows) stimulate both tumor stroma cells, including tumor-associated fibroblasts, and adjacent adipose tissue to increase ATX production. The tumor also recruits other circulating cells including macrophages to further increase inflammatory signaling and promote a pro-survival and pro-growth environment. Increased ATX enzymatic activity increases tumor LPA concentrations (green arrows), which, thereby, initiates a vicious cycle that further fuels tumor growth and ultimately metastasis.

It was concluded from this combined work that adipocytes and fibroblasts in the proximity of the cancer cells are stimulated by inflammatory cytokines produced by the tumors and that this increases ATX synthesis and secretion [9,23,67] (Figure 4). This amplifies the inflammatory cycle and promotes accumulation of inflammatory macrophages [44,45]. This evidence for bi-directional signaling between breast tumors and adipose tissue through the ATX-LPA-inflammatory cycle has since been confirmed by others [73,74].

## 4. Influence of Obesity on Breast Cancer

Inflamed adipose tissue is one of the characteristics of obesity that causes a chronic low-grade systemic inflammation [75]. Circulating levels of interleukin-6 (IL-6) and tumor necrosis factor-alpha (TNF-α) increase in obese individuals, and adipose tissue is a major source of these pro-inflammatory cytokines [76,77]. Inflamed adipocytes also secrete monocyte chemoattractant protein) (MCP-1), which increases macrophage infiltration [78]. These recruited macrophages shift to a pro-inflammatory phenotype that exacerbates the inflammation [79]. There is good evidence that obesity is associated with the development of premenopausal triple-negative breast cancer [80]. In patients with confirmed breast cancers, obesity also increases the risk of metastasis, tumor recurrence, and mortality [81,82].

The impact of obesity on breast cancer is likely mediated through inflammation [83]. Overexpression MCP-1 in the mammary fat pad generates an obese-like microenvironment, which promotes breast tumor formation by increasing macrophage infiltration and angiogenesis in the adipose tissue. Induction of obesity by feeding a high-fat diet in the mouse mammary tumor virus (MMTV)-PyMT mouse model of spontaneous breast cancer causes mammary adipose tissue inflammation with elevated macrophage recruitment and angiogenesis [84]. In addition, higher levels of IL-6 production in adipose tissue associated with human breast tumors were associated with a larger tumor size and more extensive lymph node involvement [85].

Inflammation in adipose tissue stimulates the production of VEGF, which is the main mediator of blood vessel formation in adipose tissue [86]. Elevated cyclooxygenase-2 (COX-2)-induced prostaglandin E2 (PGE2) formation in inflamed adipose tissue increases the risk of breast cancer in obese women by inducing activity of aromatase in mammary adipose tissue, which increases the conversion of circulating androgens to estrogens [87]. Inflammation also increases the immune-suppressive CD4+ regulatory T cells in human subcutaneous adipose tissue [88].

Studies on mice without cancer, but with an adipocyte-specific knock out of ATX, show that ~35% of the body’s ATX is produced by adipocytes. This adipocyte contribution to ATX production increases when feeding a high fat human type diet [89,90]. ATX production increases in obesity, especially when adipose tissue is inflamed [8,44]. Serum ATX levels and the concentrations of 16:0-LPA correlate with the body mass index and waist circumference in older overweight and obese patients, even though these findings are not consistent over all populations examined [91]. Inflamed adipose tissue increases the co-morbidities of insulin resistance, diabetes, dyslipidemia, hypertension, and atherosclerosis. These conditions are characterized by low concentrations of plasma adiponectin [92] and LPA decreases adiponectin secretion [89]. There is an association of obesity with ~30% of breast cancers [93,94] and increased ATX secretion from adipocytes could contribute to this relationship. Secreted ATX binds to integrins and syndecan-4 on the surface of neighboring cells, including cancer cells. This binding appears to selectively channel LPA signaling to LPA receptors [68,69,70,71], which could be an important part of cancer progression.

Increasing the expressions of ATX or LPA1–3 receptors in mammary epithelial cells increased the development of spontaneous breast tumors in mice [95]. Increased expressions of ATX in stroma cells and LPA3 receptors in epithelial cells are associated with the aggressiveness of human breast cancer in women [72]. ATX levels correlate with tumor invasiveness [8,10,52] and the ATX gene (*ENPP2*) is one of the 40–50 most up-regulated genes in metastatic tumors [53,54,55]. We showed that inhibiting ATX activity in a syngeneic mouse model of breast cancer decreased breast tumor growth and metastasis to the lungs in mice [14].

Immunohistochemical analysis of tissues from breast cancer patients shows that inflammatory stroma, which expresses higher LPA3, is more likely to have a higher histological grade, and more frequent estrogen receptor (ER)/progesterone receptor (PR) and human epidermal growth factor receptor 2 (HER2) positivity, which, overall, is associated with a worse phenotype [96]. LPA2 and LPA3 receptors were highly expressed in human breast cancer cells and in stromal cells, respectively, when the stroma contained >50% of adipose tissue [96]. In addition, in breast tumors with adipose tissue-rich stroma, the number of CD163+ macrophages was greater with stromal ATX positivity, and the numbers of CD68-positive and CD163-positive macrophages were greater in cases with stromal LPA3 receptor positivity. LPA3 receptor expression in stromal cells from adipose tissue-rich stroma was positively correlated with a shorter disease-free survival. It was concluded that ATX-LPA signaling proteins are highly expressed in breast tumors with adipose tissue-rich stroma and this is associated with macrophage infiltration and poorer patient outcomes [96].

## 5. Role of Lipid Phosphate Phosphatases in Controlling LPA Signaling and Tumor Progression

Another important component in the regulation of LPA signaling in cancers is the degradation of LPA by LPP1 and LPP3 (*PLPP1* and *PLPP3*, respectively). The expressions of these two LPPs are decreased in lung, ovarian, and breast tumors [97,98,99] and this can contribute to the increase of LPA concentrations in the tumors [100,101,102]. Low expression of mRNA for LPP1 is one of 12 changes in mRNA that predicts poor survival in breast cancer patients [103]. We showed that mRNA concentrations for LPP1 were lower in all types of breast tumors compared to normal breast tissue [27,51].

The consequence of low LPP1 activity has a two-fold implication. First, there is less degradation of LPA in the vicinity of the tumors. Second, expression of LPP1 attenuates signaling downstream of LPA and protease activated receptors and this is not related to the dephosphorylation of extracellular LPA. The LPPs are expressed on internal membranes as well as on plasma membranes where they dephosphorylate bioactive lipid phosphates and pyrophosphates including phosphatidate and ceramide 1-phosphate, which are involved in intracellular signaling [21,104,105]. This could explain why increasing LPP1 activity attenuates the activation of Ca^2+^-transients [106] and phosphatidate accumulation after phospholipase D activation [107]. These presumed intracellular actions are downstream of receptor activation because LPP1 attenuates the effects of an LPA analogue (wls-31) that activates LPA receptors, but cannot be dephosphorylated [106,107]. Furthermore, LPP1 attenuates signaling by the protease-activated receptor-1 (PAR1) in MDA-MB-231 breast cancer cells [106]. This latter effect of LPP1 requires phosphatase activity and it cannot be explained by dephosphorylation of extracellular LPA. Thrombin-induced ERK phosphorylation is also inhibited by LPP1 [108]. One interpretation of these results is that the LPPs dephosphorylate a lipid mediator that facilitates signaling downstream of the activation of several different types of GPCRs [21,104]. Thus, the low activity of LPP1 in cancer cells makes them hypersensitive to the effects of ATX and signaling by LPA [10].

Increasing the low levels of LPP1 in breast cancer cells decreases cell division and blocks tumor growth and metastasis in a mouse breast cancer model by up to 80% [27,106]. The low expression of LPP1 in cancer cells is associated with increased expression of the metalloproteinases, (MMP)-1, 3, 7, 9, 10, 12, 13 and cyclin D1/D3, which are transcribed downstream of the AP1 (Fos-Jun) complex and the tumors have increased expression of c-Fos and c-Jun [27]. The increased expression of MMPs is associated with decreased collagen content in the tumors of experimental mice. This low collagen content could allow cancer cells to exit from the breast tumor, enter the circulation, and metastasize to other organs. Breast tumors from patients also show increased expression of MMP-1, 7, 8, 9, 12, 13 and the tumors have increased expression of c-Fos and c-Jun. This could contribute to increased metastasis and explain why breast cancer patients that have low expression of LPP1 in their tumors exhibit increased mortality [27]. This relationship was not statistically significant for LPP3 in breast cancer patients [27] even though increasing LPP3 expression in mouse ovarian cancer cells attenuates their ability to support tumor growth [106,109].

In contrast to this, LPP2 has a completely different effect and mRNA concentrations for LPP2 (PLPP2) are increased in breast, lung, and ovarian tumors [21]. A genomic screen between normal and transformed mesenchymal stem cells showed that LPP2 is elevated in several cancer cell lines including MCF7, SK-LMS1, MG63, and U2OS cells [110]. We showed that increasing LPP2 expression in fibroblasts stimulates the cell division [111]. Increased LPP2 expression in cancer cells is part of the transformed phenotype and it facilitates anchorage-dependent cell growth [110]. Our unpublished work shows that knockout of LPP2 in MDA-MB-231 breast cancer cells decreases tumor growth by ~70% in a mouse breast cancer model. Although increased LPP2 activity increases the degradation of extracellular LPA, which would decrease tumor progression, the selective effects of LPP2 on intracellular signaling likely account for its negative effects on tumor growth.

## 6. Role of Adipose Tissue-Derived ATX in Responses of Breast Tumors to Chemotherapy

**Figure 5:**
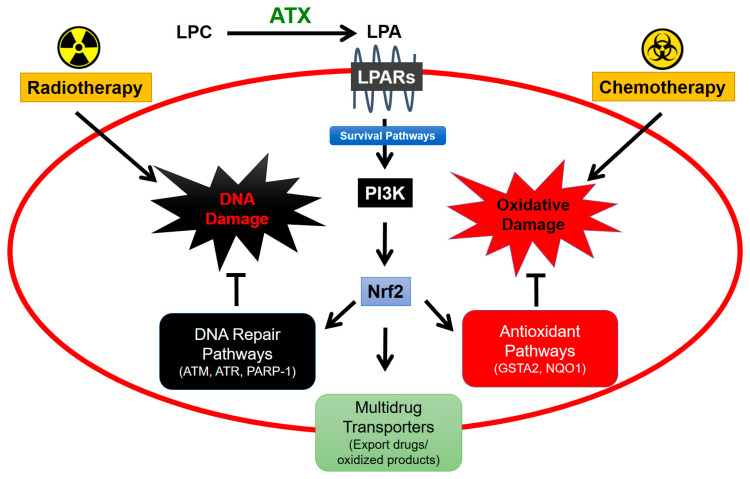
ATX signaling protects cancers cells from cytotoxic effects of radiotherapy and chemotherapy. Lysophosphatidate (LPA) signaling stabilizes Nrf2 expression via PI3K-mediated survival pathways [115]. Nrf2 facilitates expression of proteins involved in DNA repair and antioxidant pathways as well as increases expression of multidrug-resistant transporters on the cancer cell surface for export of drug and oxidized molecules from the cell [29]. Combined, these mechanisms contribute to cancer cell survival and resistance to cancer therapy.

Adipose tissue plays an important role in breast cancer and its treatment because adipocytes are the major source of ATX activity in the breast. The secretion of ATX is increased further by the inflammatory signals that the adipocytes receive from cytokines/chemokines secreted by cancer cells, stromal cells, and leukocytes. The survival signals generated by ATX through subsequent signaling through LPA receptors on tumor-associated cells decrease the efficacies of pacitaxel [65], tamoxifen [112], and doxorubicin [113] in killing the breast cancer cells. These are major therapeutics for different types of breast cancer. Axiomatically, inhibiting ATX activity increased the efficacy of doxorubicin in decreasing breast tumor growth and metastasis in mice [114]. This action is indirect and occurs because of decreased LPA production and activation of LPA1 receptors and phosphoinositide 3-kinase (PI3K). This signaling stabilizes the transcription factor, nuclear factor erythroid-derived 2-like 2 (Nrf2), which activates the anti-oxidant response element, and leads to the synthesis of anti-oxidant proteins and multi-drug resistance transporters [113] (Figure 5). These changes protect cancer cells by decreasing oxidative damage and by exporting chemotherapeutic drugs and toxic oxidation products.

## 7. Role of Adipose Tissue-Derived ATX in Responses to Radiotherapy for Breast Cancer

In the treatment of breast cancer, ~60% of patients have their tumors removed surgically (lumpectomy). This is followed by radiotherapy (RT) to the post-surgical breast with daily fractions of 1.8–2 Gy/fraction to a total dose of 45–50 Gy. The most recent American Society for Radiation Oncology (ASTRO) guidelines for women with invasive breast cancer recommend hypo-fractionated RT with either 40 Gy in 15 fractions or 42.5 Gy in 16 fractions, i.e., ~2.6 Gy per fraction [116]. This creates a fairly unique situation in which breast adipose tissue is irradiated and damaged multiple times.

We, therefore, investigated the effects of radiation-induced damage to adipose tissue. Exposure of cultured human breast adipose tissue to 0.5 to 5 Gy of γ-radiation increased the production of ATX as well as the LPA signaling through increased expression of LPA1 and LPA2 receptors downstream of DNA damage [117]. These events caused activation of a feed-forward inflammatory cycle including an increase in the expression of COX-2 and multiple inflammatory cytokines/chemokines, which, in turn, increase more ATX secretion [44,45]. The inflammatory cycle could be attenuated by inhibiting the RT-induced activation of ATM, poly [ADP-ribose] polymerase-1 (PARP-1), and NFκB.

This work in vitro was extended in vivo using precision RT on a mammary fat pad in mice with a small-animal “image-guided” RT platform (SARRP) with integrated computed tomography (CT)-imaging. This system allows for treatment-planned RT to the tumor and fat pad while minimizing peripheral tissue damage. In these studies, a single dose of RT increased plasma ATX concentrations [118]. There was no significant effect of one dose of RT on plasma concentrations of IL-6 and TNF-α, but three fractions of RT substantially increased these inflammatory cytokines [118]. A similar increase was observed after three fractions of RT for VEGF, G-CSF, CCL11, and CXCL10 in the irradiated adipose tissue [118]. These effects of multiple fractions of RT likely depend on the cumulative DNA and tissue damage. In addition, repeated doses of RT increase Nrf2 expression [118], which raises the synthesis of numerous proteins that attenuate oxidative damage and promote DNA repair [119,120] (Figure 5). Previous work predicted that Nrf2 blockade could provide a target for increasing the efficacy of RT by attenuating DNA repair [121]. The results indicate that RT-induced expression of ATX appears to be an early event in vivo following RT-induced damage and that subsequent LPA signaling could then augment the release of inflammatory cytokines and chemokines to produce an inflammatory response.

LPA also decreases adiponectin secretion [89]. Secretion of adiponectin by adipose tissue normally produces an anti-inflammatory response and it is inversely linked to the risk of obesity-associated malignancies and insulin resistance [122]. Plasma adiponectin levels were decreased significantly by three fractions of RT in non-tumor bearing mice [118] and this could also be a response to increased LPA signaling. Plasma adiponectin was also significantly lower in tumor-bearing mice and this level was not decreased further by RT. Three fractions of RT appeared to decrease adiponectin levels in both normal and tumor-associated adipose tissue. However, the effect only reached statistical significance in the latter instance. The leptin/adiponectin ratio was increased in tumor-bearing mice, but RT had no effect on this ratio in either tumor-bearing or control mice [118].

Tumor-bearing mice showed substantially decreased plasma levels of amylin, which is co-secreted with insulin by pancreatic β-cells [118]. However, these levels were unaffected by RT in control and tumor-bearing mice. The plasma concentrations of hormones, which maintain glucose homeostasis and regulation of body weight, including insulin, glucagon, glucagon-like peptide, ghrelin, and pancreatic polypeptide, were not significantly lower in tumor-bearing versus control mice and these were not altered significantly by RT in either case.

Repeated activation of the ATX-LPA-inflammatory cycle should decrease the efficacy of RT by stimulating a wound healing response [11,123,124]. First, RT-induced increases in expressions of ATX and activation of LPA2 receptors could decrease cancer cell death by depletion of the pro-apoptotic protein, Siva-1 [11]. However, RT-induced apoptosis is less important in solid tumors compared to the effects on intestinal epithelial cells. The major therapeutic effect of RT for breast tumors is in causing some form of cytostasis (senescence or polyploid giant-cell formation) [125]. In agreement with this, administration of the ATX inhibitor, GLPG1690, at the time of irradiation with five fractions of 7.5 Gy to breast tumors in mice decreased proliferation of breast cancer cells in the remaining tumor following the irradiation [126]. This work supports the hypothesis that blocking the RT-induced activation of the ATX-LPA-inflammatory cycle can improve the efficacy of RT in eliminating residual cancer cells.

## 8. Role of the Anti-Inflammatory Glucocorticoid, Dexamethasone (DEX), in Attenuating ATX and LPA Signaling in Adipose Tissue

We determined whether the anti-inflammatory glucocorticoid, DEX, could be used to decrease signaling through the ATX-LPA-inflammatory cycle by using cultured rat and human adipose tissue as an experimental model because adipocytes are a major site of ATX secretion [89,90]. DEX (10–1000 nM) decreased ATX secretion and increased LPP1 expression, which would attenuate LPA signaling, and decreased mRNA expressions for IL-6, TNF-α, PPARγ, and adiponectin [28]. Co-treatment with rosiglitazone, an insulin sensitizer, and/or insulin attenuated the DEX-induced decreases in ATX and adiponectin secretion, but did not reverse DEX-induced decreases in secretions of 20 inflammatory cytokines/chemokines [28]. DEX-treated mice exhibited lower ATX activity in plasma, brain, and adipose tissue, decreased mRNA levels for LPA1/2 receptors in the brain, and decreased plasma concentrations of LPA.

We were concerned that DEX, a glucocorticoid, would contribute to the co-morbidities of the Metabolic Syndrome (insulin resistance, hyperglycemia, dyslipidemias, etc.) since the natural glucocorticoid, cortisol, has this effect [127]. First, cortisol opposes many of the pathways that are stimulated by insulin such as in glycolysis versus gluconeogenesis, hepatic glucose release, and glucose uptake in muscle and adipose tissue. By contrast, glucocorticoids augment the actions of insulin in stimulating energy deposition through glycogen, fatty acid synthesis, and the uptake of fatty acids by adipose tissue through the actions of lipoprotein lipase [127,128]. These adverse effects were indicated by the DEX-induced decreases in the expression of adiponectin and the increase in leptin seen in adipose tissue cultures [28]. However, these potentially adverse changes in the adiponectin: leptin ratios were not seen in mice that were treated for four days with DEX [28]. Despite this, plasma ATX activity was decreased and the levels of COX-2 and LPA2 receptors in mammary adipose tissue were decreased. A possible beneficial effect of DEX in this mouse model was the decrease in the expression of inflammatory cytokines and adipose tissue inflammation [28,128], which can, otherwise, lead to insulin insensitivity in adipose tissue and adjacent muscles.

## 9. Effects of DEX on RT-Induced Secretion of ATX and Subsequent LPA Signaling on RT-Induced Fibrosis

Radiotherapy is a mainstay of cancer treatment. However, one of the major side effects of RT is the development of fibrosis. This also has a component of LPA signaling. Signaling through the ATX-LPA-LPA1 receptor pathway is widely recognized as a major contributor to various types of fibrotic pathologies, especially in the liver and lungs [129,130,131,132,133,134,135,136,137,138,139,140]. Inhibition of ATX and LPA signaling attenuates lung fibrosis, including in idiopathic pulmonary fibrosis (IPF) and the fibrosis that occurs following exposure to bleomycin [139,141,142], which is a radiomimetic anticancer agent [143]. The importance of this pathway in fibrosis is supported by clinical trials in which drugs such as GLPG1690 (an ATX inhibitor) and BMS986020 (an LPA1 receptor antagonist) attenuated the progression of IPF [144,145]. LPA-mediated pro-inflammatory signaling is very likely to be a driving force in RT-induced fibrosis. However, it has not yet been established.

We, therefore, used normal Balb/c mice and the orthotopic 4T1-Balb/c mouse syngeneic model of breast cancer to study how the effects of DEX on LPA signaling could modify RT-induced fibrosis. DEX treatment during fractionated RT attenuated fibrosis in the irradiated fat pad of Balb/c mice by ~70% at 7 weeks following the irradiation [146]. DEX treatment decreased plasma ATX activity at 2 days after 3 fractions of RT by ~43% in normal mice, but ATX activity in the plasma returned to non-DEX treatment levels at 7 weeks after 5 fractions of RT. Total ATX activities in the irradiated fat pad and lungs were also not changed significantly after seven weeks following treatment with DEX [146]. Despite this, there was a change in the distribution of ATX in the adipose tissue and lungs. ATX was evenly distributed in the interstitium of adipose tissue in both the control mammary fat pad at two days post-IR. The distribution pattern of ATX was altered at seven weeks after RT when ATX was preferentially localized around blood vessels. This distribution reflected that of fibrous tissue in fat pads and lungs. This association of ATX with blood vessels was attenuated by DEX along with the decreased fibrotic response [146]. ATX-LPA signaling through LPA1 receptors is critical for vascular development [147] and vasculitis [148,149]. Increased LPA signaling is also associated with injury to the vascular endothelium, including the increased vascular permeability and leakage associated with bleomycin-induced lung fibrosis in mice. In this case, inhibiting signaling through LPA1 receptors attenuated both vascular injury and fibrosis [141,150,151,152].

We also determined the interaction of RT with DEX on adiponectin secretion since this could indicate an adverse effect of metabolism. DEX treatment with RT did not significantly alter the adiponectin content of the plasma or in the adipose tissue as compared to RT alone in both mouse models. DEX treatment surprisingly decreased the plasma glucose concentrations in non-tumor bearing mice (10.4 ± 0.4 mM versus 7.8 ± 0.3 mM; *p* = 0.002), while not causing any change of glucose levels in tumor-bearing mice (7.7 ± 0.33 mM vs. 6.4 ± 0.4 mM).

This combined work demonstrates that inhibiting RT-induced activation of the ATX-LPA-inflammatory cycle is a potential strategy for decreasing RT-induced breast fibrosis. Furthermore, DEX attenuated inflammation and fibrosis in the lungs, as expected, based on work by other investigators [153,154,155]. However, our work links this activity of DEX for the first time to its ability to attenuate LPA signaling, which is now acknowledged to be a major stimulus for the progression of fibrosis. There are two major disadvantages of using DEX to decrease RT-induced fibrosis, which mitigate against its acceptance as a therapy for this condition. First, DEX can decrease the sensitivity of the immune system. Secondly, the therapeutic effects of DEX in decreasing lung fibrosis were found not to be temporally robust in previous work [154,156,157]. Thus, it will likely be far more advantageous to target the ATX-LPA-inflammatory axis more specifically and directly [146].

## 10. Future Perspective

Although there is no existing approach to targeting ATX-LPA axis for cancer treatment, there is now sufficient evidence to establish that LPA is a significant inducer that increases tumor growth, metastasis, and loss of efficacy of chemotherapy and RT. Blocking LPA formation and signaling as part of cancer treatment has become possible because at least three inhibitors against LPA signaling have proven to be safe in clinical trials [144,145,158]. GLPG1690, which is an ATX inhibitor, is currently in a Phase 3 trial for IPF [144].

We studied two ATX inhibitors, ONO-8430506 and GLPG1690, in a syngeneic mouse model of triple negative breast cancer [14,115]. Blocking ATX activity increased the antitumor efficacy of chemotherapy and RT in combination treatments. This effect is likely to be independent of breast cancer type because most of the ATX is not derived from breast cancer cells. The use of ATX inhibitors could, therefore, be particularly important in triple negative breast cancer for which there are fewer targeted therapeutic options.

In terms of the LPA receptor antagonists, BMS-986020 (LPA1 receptor antagonist) and SAR100842 (LPA1/3 receptors antagonist), have completed Phase 2 trials for IPF and systemic sclerosis, respectively [145,158]. Such compounds could be readily introduced into clinical trials to test their efficacies as adjuvant treatments for cancer. Inhibiting ATX activity by about 80% in vivo has the potential advantage that it could attenuate the activation of all LPA receptors, especially LPA1, LPA2, and LPA3 receptors. However, such global inhibition could impact other physiological activities that require LPA mediated signaling. For example, it may not be beneficial to block LPA4 and LPA5 receptors, which have inhibitory effects on cancer cell growth and motility. On the other hand, blocking the activation of LPA5 receptors could theoretically decrease the ability of cancer cells to evade the immune system. The effects of ATX inhibitors and LPA receptor antagonists in improving the different types of immune therapy and the use of checkpoint inhibitors has received relatively little attention and this could be a fruitful area of cancer research.

We are at an exciting point where introducing novel agents that can decrease the activation of the ATX-LPA-inflammatory cycle could provide valuable adjuvant therapies for improving the treatments of cancer patients.

## Abbreviations

ATX	autotaxin
COX-1,2	cyclooxygenase-1,2
IL	interleukin
IPF	idiopathic pulmonary fibrosis
LPA	lysophosphatidate at physiological pH but often referred to as lysophosphatidic acid
LPC	lysophosphatidylcholine
LPA1–6	lysophosphatidate 1–6 receptors
LPP	lipid phosphate phosphatase
NFκB	nuclear factor kappa-light-chain-enhancer of activated B cells
Nrf2	nuclear erythroid 2-like 2
RT	radiotherapy
S1P	sphingosine 1-phosphate
TNF-α	tumor necrosis factor-α
VEGF	vascular endothelial growth factor

## References

[1] Murata J., Lee H.Y., Clair T., Krutzsch H.C., Arestad A.A., Sobel M.E., Liotta L.A., Stracke M.L. (1994). Cdna cloning of the human tumor motility-stimulating protein, autotaxin, reveals a homology with phosphodiesterases. J. Biol. Chem..

[2] Stefan C., Jansen S., Bollen M. (2005). Npp-type ectophosphodiesterases: Unity in diversity. Trends Biochem. Sci..

[3] Perrakis A., Moolenaar W.H. (2014). Autotaxin: Structure-function and signaling. J. Lipid Res..

[4] Stracke M.L., Krutzsch H.C., Unsworth E.J., Arestad A., Cioce V., Schiffmann E., Liotta L.A. (1992). Identification, purification, and partial sequence analysis of autotaxin, a novel motility-stimulating protein. J. Biol. Chem..

[5] Umezu-Goto M., Kishi Y., Taira A., Hama K., Dohmae N., Takio K., Yamori T., Mills G.B., Inoue K., Aoki J. (2002). Autotaxin has lysophospholipase d activity leading to tumor cell growth and motility by lysophosphatidic acid production. J. Cell Biol..

[6] Tokumura A., Majima E., Kariya Y., Tominaga K., Kogure K., Yasuda K., Fukuzawa K. (2002). Identification of human plasma lysophospholipase d, a lysophosphatidic acid-producing enzyme, as autotaxin, a multifunctional phosphodiesterase. J. Biol. Chem..

[7] Gaetano C.G., Samadi N., Tomsig J.L., Macdonald T.L., Lynch K.R., Brindley D.N. (2009). Inhibition of autotaxin production or activity blocks lysophosphatidylcholine-induced migration of human breast cancer and melanoma cells. Mol. Carcinog..

[8] Benesch M.G.K., Ko Y.M., McMullen T.P.W., Brindley D.N. (2014). Autotaxin in the crosshairs: Taking aim at cancer and other inflammatory conditions. FEBS Lett..

[9] Hemmings D.G., Brindley D.N. (2020). Signalling by lysophosphatidate and its health implications. Essays Biochem..

[10] Samadi N., Bekele R., Capatos D., Venkatraman G., Sariahmetoglu M., Brindley D.N. (2011). Regulation of lysophosphatidate signaling by autotaxin and lipid phosphate phosphatases with respect to tumor progression, angiogenesis, metastasis and chemo-resistance. Biochimie.

[11] Brindley D.N., Lin F.T., Tigyi G.J. (2013). Role of the autotaxin-lysophosphatidate axis in cancer resistance to chemotherapy and radiotherapy. Biochim. Biophys. Acta.

[12] Van Meeteren L.A., Ruurs P., Stortelers C., Bouwman P., van Rooijen M.A., Pradere J.P., Pettit T.R., Wakelam M.J., Saulnier-Blache J.S., Mummery C.L. (2006). Autotaxin, a secreted lysophospholipase d, is essential for blood vessel formation during development. Mol. Cell. Biol..

[13] Pamuklar Z., Federico L., Liu S., Umezu-Goto M., Dong A., Panchatcharam M., Fulkerson Z., Berdyshev E., Natarajan V., Fang X. (2009). Autotaxin/lysopholipase d and lysophosphatidic acid regulate murine hemostasis and thrombosis. J. Biol. Chem..

[14] Benesch M.G.K., Tang X., Maeda T., Ohhata A., Zhao Y.Y., Kok B.P.C., Dewald J., Hitt M., Curtis J.M., McMullen T.P.W. (2014). Inhibition of autotaxin delays breast tumor growth and lung metastasis in mice. FASEB J..

[15] Albers H.M., Dong A., van Meeteren L.A., Egan D.A., Sunkara M., van Tilburg E.W., Schuurman K., van Tellingen O., Morris A.J., Smyth S.S. (2010). Boronic acid-based inhibitor of autotaxin reveals rapid turnover of lpa in the circulation. Proc. Natl. Acad. Sci. USA.

[16] Koike S., Yutoh Y., Keino-Masu K., Noji S., Masu M., Ohuchi H. (2011). Autotaxin is required for the cranial neural tube closure and establishment of the midbrain-hindbrain boundary during mouse development. Dev. Dyn..

[17] Hemrika W., Renirie R., Dekker H.L., Barnett P., Wever R. (1997). From phosphatases to vanadium peroxidases: A similar architecture of the active site. Proc. Natl. Acad. Sci. USA.

[18] Neuwald A.F. (1997). An unexpected structural relationship between integral membrane phosphatases and soluble haloperoxidases. Protein Sci..

[19] Brindley D.N., English D., Pilquil C., Buri K., Ling Z.C. (2002). Lipid phosphate phosphatases regulate signal transduction through glycerolipids and sphingolipids. Biochim. Biophys. Acta.

[20] Carman G.M., Han G.S. (2009). Phosphatidic acid phosphatase, a key enzyme in the regulation of lipid synthesis. J. Biol. Chem..

[21] Tang X., Benesch M.G., Brindley D.N. (2015). Lipid phosphate phosphatases and their roles in mammalian physiology and pathology. J. Lipid Res..

[22] Van Meeteren L.A., Ruurs P., Christodoulou E., Goding J.W., Takakusa H., Kikuchi K., Perrakis A., Nagano T., Moolenaar W.H. (2005). Inhibition of autotaxin by lysophosphatidic acid and sphingosine 1-phosphate. J. Biol. Chem..

[23] Benesch M.G.K., Zhao Y.Y., Curtis J.M., McMullen T.P., Brindley D.N. (2015). Regulation of autotaxin expression and secretion by lysophosphatidate and sphingosine 1-phosphate. J. Lipid Res..

[24] Jansen S., Andries M., Vekemans K., Vanbilloen H., Verbruggen A., Bollen M. (2009). Rapid clearance of the circulating metastatic factor autotaxin by the scavenger receptors of liver sinusoidal endothelial cells. Cancer Lett..

[25] Tang X., Zhao Y.Y., Dewald J., Curtis J.M., Brindley D.N. (2016). Tetracyclines increase lipid phosphate phosphatase expression on plasma membranes and turnover of plasma lysophosphatidate. J. Lipid Res..

[26] Salous A.K., Panchatcharam M., Sunkara M., Mueller P., Dong A., Wang Y., Graf G.A., Smyth S.S., Morris A.J. (2013). Mechanism of rapid elimination of lysophosphatidic acid and related lipids from the circulation of mice. J. Lipid Res..

[27] Tang X., McMullen T.P.W., Brindley D.N. (2019). Increasing the low lipid phosphate phosphatase 1 activity in breast cancer cells decreases transcription by ap-1 and expressions of matrix metalloproteinases and cyclin d1/d3. Theranostics.

[28] Meng G., Tang X., Yang Z., Zhao Y., Curtis J.M., McMullen T.P.W., Brindley D.N. (2019). Dexamethasone decreases the autotaxin-lysophosphatidate-inflammatory axis in adipose tissue: Implications for the metabolic syndrome and breast cancer. FASEB J..

[29] Benesch M.G.K., Tang X., Brindley D.N. (2020). Autotaxin and breast cancer: Towards overcoming treatment barriers and sequelae. Cancers.

[30] Ninou I., Magkrioti C., Aidinis V. (2018). Autotaxin in pathophysiology and pulmonary fibrosis. Front. Med..

[31] Van Corven E.J., van Rijswijk A., Jalink K., van der Bend R.L., van Blitterswijk W.J., Moolenaar W.H. (1992). Mitogenic action of lysophosphatidic acid and phosphatidic acid on fibroblasts. Dependence on acyl-chain length and inhibition by suramin. Biochem. J..

[32] Chabaud S., Marcoux T.L., Deschênes-Rompré M.P., Rousseau A., Morissette A., Bouhout S., Bernard G., Bolduc S. (2015). Lysophosphatidic acid enhances collagen deposition and matrix thickening in engineered tissue. J. Tissue Eng. Regen. Med..

[33] Van Corven E.J., Groenink A., Jalink K., Eichholtz T., Moolenaar W.H. (1989). Lysophosphatidate-induced cell proliferation: Identification and dissection of signaling pathways mediated by g proteins. Cell.

[34] Panetti T.S., Chen H., Misenheimer T.M., Getzler S.B., Mosher D.F. (1997). Endothelial cell mitogenesis induced by lpa: Inhibition by thrombospondin-1 and thrombospondin-2. J. Lab. Clin. Med..

[35] Knowlden S., Georas S.N. (2014). The autotaxin-lpa axis emerges as a novel regulator of lymphocyte homing and inflammation. J. Immunol..

[36] Ray R., Rai V. (2017). Lysophosphatidic acid converts monocytes into macrophages in both mice and humans. Blood.

[37] Mazereeuw-Hautier J., Gres S., Fanguin M., Cariven C., Fauvel J., Perret B., Chap H., Salles J.P., Saulnier-Blache J.S. (2005). Production of lysophosphatidic acid in blister fluid: Involvement of a lysophospholipase d activity. J. Investig. Dermatol..

[38] Magkrioti C., Galaris A., Kanellopoulou P., Stylianaki E.A., Kaffe E., Aidinis V. (2019). Autotaxin and chronic inflammatory diseases. J. Autoimmun..

[39] Dvorak H.F. (1986). Tumors: Wounds that do not heal. Similarities between tumor stroma generation and wound healing. N. Engl. J. Med..

[40] Schafer M., Werner S. (2008). Cancer as an overhealing wound: An old hypothesis revisited. Nat. Rev. Mol. Cell Biol..

[41] Hanahan D., Weinberg R.A. (2011). Hallmarks of cancer: The next generation. Cell.

[42] Tigyi G.J., Yue J., Norman D.D., Szabo E., Balogh A., Balazs L., Zhao G., Lee S.C. (2019). Regulation of tumor cell—Microenvironment interaction by the autotaxin-lysophosphatidic acid receptor axis. Adv. Biol. Regul..

[43] Colotta F., Allavena P., Sica A., Garlanda C., Mantovani A. (2009). Cancer-related inflammation, the seventh hallmark of cancer: Links to genetic instability. Carcinogenesis.

[44] Benesch M.G.K., MacIntyre I.T.K., McMullen T.P.W., Brindley D.N. (2018). Coming of age for autotaxin and lysophosphatidate signaling: Clinical applications for preventing, detecting and targeting tumor-promoting inflammation. Cancers.

[45] Benesch M.G.K., Yang Z., Tang X., Meng G., Brindley D.N. (2017). Lysophosphatidate signaling: The tumor microenvironment’s new nemesis. Trends Cancer.

[46] Lagadari M., Truta-Feles K., Lehmann K., Berod L., Ziemer M., Idzko M., Barz D., Kamradt T., Maghazachi A.A., Norgauer J. (2009). Lysophosphatidic acid inhibits the cytotoxic activity of nk cells: Involvement of gs protein-mediated signaling. Int. Immunol..

[47] Mathew D., Kremer K.N., Strauch P., Tigyi G., Pelanda R., Torres R.M. (2019). Lpa5 is an inhibitory receptor that suppresses cd8 t-cell cytotoxic function via disruption of early tcr signaling. Front. Immunol..

[48] Smyth S.S., Mueller P., Yang F., Brandon J.A., Morris A.J. (2014). Arguing the case for the autotaxin-lysophosphatidic acid-lipid phosphate phosphatase 3-signaling nexus in the development and complications of atherosclerosis. Arterioscler. Thromb. Vasc. Biol..

[49] Yang B., Zhou Z., Li X., Niu J. (2016). The effect of lysophosphatidic acid on toll-like receptor 4 expression and the nuclear factor-κb signaling pathway in thp-1 cells. Mol. Cell. Biochem..

[50] So J., Wang F.Q., Navari J., Schreher J., Fishman D.A. (2005). Lpa-induced epithelial ovarian cancer (eoc) in vitro invasion and migration are mediated by vegf receptor-2 (VEGF-R2). Gynecol. Oncol..

[51] Tang X., Benesch M.G.K., Brindley D.N. (2020). Role of the autotaxin-lysophosphatidate axis in the development of resistance to cancer therapy. Biochim. Biophys. Acta Mol. Cell Biol. Lipids.

[52] St-Coeur P.D., Ferguson D., Morin P., Touaibia M. (2013). Pf-8380 and closely related analogs: Synthesis and structure-activity relationship towards autotaxin inhibition and glioma cell viability. Arch. Pharm..

[53] Euer N., Schwirzke M., Evtimova V., Burtscher H., Jarsch M., Tarin D., Weidle U.H. (2002). Identification of genes associated with metastasis of mammary carcinoma in metastatic versus non-metastatic cell lines. Anticancer Res..

[54] Castellana B., Escuin D., Peiro G., Garcia-Valdecasas B., Vazquez T., Pons C., Perez-Olabarria M., Barnadas A., Lerma E. (2012). Aspn and gjb2 are implicated in the mechanisms of invasion of ductal breast carcinomas. J. Cancer.

[55] Vital A.L., Tabernero M.D., Castrillo A., Rebelo O., Tao H., Gomes F., Nieto A.B., Resende Oliveira C., Lopes M.C., Orfao A. (2010). Gene expression profiles of human glioblastomas are associated with both tumor cytogenetics and histopathology. Neuro Oncol..

[56] Funes S.C., Rios M., Escobar-Vera J., Kalergis A.M. (2018). Implications of macrophage polarization in autoimmunity. Immunology.

[57] Italiani P., Boraschi D. (2014). From monocytes to M1/M2 macrophages: Phenotypical vs. Functional differentiation. Front. Immunol..

[58] Ohashi K., Parker J.L., Ouchi N., Higuchi A., Vita J.A., Gokce N., Pedersen A.A., Kalthoff C., Tullin S., Sams A. (2010). Adiponectin promotes macrophage polarization toward an anti-inflammatory phenotype. J. Biol. Chem..

[59] Reinartz S., Lieber S., Pesek J., Brandt D.T., Asafova A., Finkernagel F., Watzer B., Nockher W.A., Nist A., Stiewe T. (2019). Cell type-selective pathways and clinical associations of lysophosphatidic acid biosynthesis and signaling in the ovarian cancer microenvironment. Mol. Oncol..

[60] Lee S.C., Dacheux M.A., Norman D.D., Balázs L., Torres R.M., Augelli-Szafran C.E., Tigyi G.J. (2020). Regulation of tumor immunity by lysophosphatidic acid. Cancers.

[61] Peng X.H., Karna P., Cao Z., Jiang B.H., Zhou M., Yang L. (2006). Cross-talk between epidermal growth factor receptor and hypoxia-inducible factor-1alpha signal pathways increases resistance to apoptosis by up-regulating survivin gene expression. J. Biol. Chem..

[62] Brindley D.N., Benesch M.G.K., Murph M.M. (2015). Autotaxin—An enzymatic augmenter of malignant progression linked to inflammation. Melanoma—Current Clinical Management and Future Therapeutics.

[63] Cerami E., Gao J., Dogrusoz U., Gross B.E., Sumer S.O., Aksoy B.A., Jacobsen A., Byrne C.J., Heuer M.L., Larsson E. (2012). The cbio cancer genomics portal: An open platform for exploring multidimensional cancer genomics data. Cancer Discov..

[64] Gao J., Aksoy B.A., Dogrusoz U., Dresdner G., Gross B., Sumer S.O., Sun Y., Jacobsen A., Sinha R., Larsson E. (2013). Integrative analysis of complex cancer genomics and clinical profiles using the cbioportal. Sci. Signal..

[65] Samadi N., Gaetano C., Goping I.S., Brindley D.N. (2009). Autotaxin protects mcf-7 breast cancer and mda-mb-435 melanoma cells against taxol-induced apoptosis. Oncogene.

[66] Yung Y.C., Stoddard N.C., Chun J. (2014). Lpa receptor signaling: Pharmacology, physiology, and pathophysiology. J. Lipid Res..

[67] Benesch M.G.K., Tang X., Dewald J., Dong W.F., Mackey J.R., Hemmings D.G., McMullen T.P., Brindley D.N. (2015). Tumor-induced inflammation in mammary adipose tissue stimulates a vicious cycle of autotaxin expression and breast cancer progression. FASEB J..

[68] Leblanc R., Sahay D., Houssin A., Machuca-Gayet I., Peyruchaud O. (2018). Autotaxin-beta interaction with the cell surface via syndecan-4 impacts on cancer cell proliferation and metastasis. Oncotarget.

[69] Leblanc R., Houssin A., Peyruchaud O. (2018). Platelets, autotaxin and lysophosphatidic acid signalling: Win-win factors for cancer metastasis. Br. J. Pharmacol..

[70] Fulkerson Z., Wu T., Sunkara M., Kooi C.V., Morris A.J., Smyth S.S. (2011). Binding of autotaxin to integrins localizes lysophosphatidic acid production to platelets and mammalian cells. J. Biol. Chem..

[71] Hausmann J., Kamtekar S., Christodoulou E., Day J.E., Wu T., Fulkerson Z., Albers H.M., van Meeteren L.A., Houben A.J., van Zeijl L. (2011). Structural basis of substrate discrimination and integrin binding by autotaxin. Nat. Struct. Mol. Biol..

[72] Popnikolov N.K., Dalwadi B.H., Thomas J.D., Johannes G.J., Imagawa W.T. (2012). Association of autotaxin and lysophosphatidic acid receptor 3 with aggressiveness of human breast carcinoma. Tumour Biol..

[73] Volden P.A., Skor M.N., Johnson M.B., Singh P., Patel F.N., McClintock M.K., Brady M.J., Conzen S.D. (2016). Mammary adipose tissue-derived lysophospholipids promote estrogen receptor-negative mammary epithelial cell proliferation. Cancer Prev. Res..

[74] Schmid R., Wolf K., Robering J.W., Strauß S., Strissel P.L., Strick R., Rübner M., Fasching P.A., Horch R.E., Kremer A.E. (2018). Adscs and adipocytes are the main producers in the autotaxin–lysophosphatidic acid axis of breast cancer and healthy mammary tissue in vitro. BMC Cancer.

[75] Fantuzzi G. (2005). Adipose tissue, adipokines, and inflammation. J. Allergy Clin. Immunol..

[76] Popko K., Gorska E., Stelmaszczyk-Emmel A., Plywaczewski R., Stoklosa A., Gorecka D., Pyrzak B., Demkow U. (2010). Proinflammatory cytokines il-6 and tnf-α and the development of inflammation in obese subjects. Eur. J. Med. Res..

[77] Bastard J.P., Jardel C., Bruckert E., Blondy P., Capeau J., Laville M., Vidal H., Hainque B. (2000). Elevated levels of interleukin 6 are reduced in serum and subcutaneous adipose tissue of obese women after weight loss. J. Clin. Endocrinol. Metab..

[78] Kanda H., Tateya S., Tamori Y., Kotani K., Hiasa K., Kitazawa R., Kitazawa S., Miyachi H., Maeda S., Egashira K. (2006). Mcp-1 contributes to macrophage infiltration into adipose tissue, insulin resistance, and hepatic steatosis in obesity. J. Clin. Investig..

[79] Thomas D., Apovian C. (2017). Macrophage functions in lean and obese adipose tissue. Metabolism.

[80] Sun H., Zou J., Chen L., Zu X., Wen G., Zhong J. (2017). Triple-negative breast cancer and its association with obesity. Mol. Clin. Oncol..

[81] Sun L., Zhu Y., Qian Q., Tang L. (2018). Body mass index and prognosis of breast cancer: An analysis by menstruation status when breast cancer diagnosis. Medicine.

[82] Blair C.K., Wiggins C.L., Nibbe A.M., Storlie C.B., Prossnitz E.R., Royce M., Lomo L.C., Hill D.A. (2019). Obesity and survival among a cohort of breast cancer patients is partially mediated by tumor characteristics. NPJ Breast Cancer.

[83] Arendt L.M., McCready J., Keller P.J., Baker D.D., Naber S.P., Seewaldt V., Kuperwasser C. (2013). Obesity promotes breast cancer by ccl2-mediated macrophage recruitment and angiogenesis. Cancer Res..

[84] Cowen S., McLaughlin S.L., Hobbs G., Coad J., Martin K.H., Olfert I.M., Vona-Davis L. (2015). High-fat, high-calorie diet enhances mammary carcinogenesis and local inflammation in mmtv-pymt mouse model of breast cancer. Cancers.

[85] Dirat B., Bochet L., Dabek M., Daviaud D., Dauvillier S., Majed B., Wang Y.Y., Meulle A., Salles B., Le Gonidec S. (2011). Cancer-associated adipocytes exhibit an activated phenotype and contribute to breast cancer invasion. Cancer Res..

[86] Tahergorabi Z., Khazaei M. (2013). The relationship between inflammatory markers, angiogenesis, and obesity. ARYA Atheroscler..

[87] Subbaramaiah K., Morris P.G., Zhou X.K., Morrow M., Du B., Giri D., Kopelovich L., Hudis C.A., Dannenberg A.J. (2012). Increased levels of cox-2 and prostaglandin e2 contribute to elevated aromatase expression in inflamed breast tissue of obese women. Cancer Discov..

[88] Zeyda M., Huber J., Prager G., Stulnig T.M. (2011). Inflammation correlates with markers of t-cell subsets including regulatory t cells in adipose tissue from obese patients. Obesity.

[89] Dusaulcy R., Rancoule C., Gres S., Wanecq E., Colom A., Guigne C., van Meeteren L.A., Moolenaar W.H., Valet P., Saulnier-Blache J.S. (2011). Adipose-specific disruption of autotaxin enhances nutritional fattening and reduces plasma lysophosphatidic acid. J. Lipid Res..

[90] Rancoule C., Dusaulcy R., Treguer K., Gres S., Guigne C., Quilliot D., Valet P., Saulnier-Blache J.S. (2012). Depot-specific regulation of autotaxin with obesity in human adipose tissue. J. Physiol. Biochem..

[91] D’Souza K., Paramel G.V., Kienesberger P.C. (2018). Lysophosphatidic acid signaling in obesity and insulin resistance. Nutrients.

[92] Lopez-Jaramillo P., Gomez-Arbelaez D., Lopez-Lopez J., Lopez-Lopez C., Martinez-Ortega J., Gomez-Rodriguez A., Triana-Cubillos S. (2014). The role of leptin/adiponectin ratio in metabolic syndrome and diabetes. Horm. Mol. Biol. Clin. Investig..

[93] Sundaram S., Johnson A.R., Makowski L. (2013). Obesity, metabolism and the microenvironment: Links to cancer. J. Carcinog..

[94] De Pergola G., Silvestris F. (2013). Obesity as a major risk factor for cancer. J. Obes..

[95] Liu S., Umezu-Goto M., Murph M., Lu Y., Liu W., Zhang F., Yu S., Stephens L.C., Cui X., Murrow G. (2009). Expression of autotaxin and lysophosphatidic acid receptors increases mammary tumorigenesis, invasion, and metastases. Cancer Cell.

[96] Cha Y.J., Koo J.S. (2019). Expression of autotaxin(-)lysophosphatidate signaling-related proteins in breast cancer with adipose stroma. Int. J. Mol. Sci..

[97] Bhattacharjee A., Richards W.G., Staunton J., Li C., Monti S., Vasa P., Ladd C., Beheshti J., Bueno R., Gillette M. (2001). Classification of human lung carcinomas by mrna expression profiling reveals distinct adenocarcinoma subclasses. Proc. Natl. Acad. Sci. USA.

[98] Curtis C., Shah S.P., Chin S.F., Turashvili G., Rueda O.M., Dunning M.J., Speed D., Lynch A.G., Samarajiwa S., Yuan Y. (2012). The genomic and transcriptomic architecture of 2,000 breast tumours reveals novel subgroups. Nature.

[99] Yoshihara K., Tajima A., Komata D., Yamamoto T., Kodama S., Fujiwara H., Suzuki M., Onishi Y., Hatae M., Sueyoshi K. (2009). Gene expression profiling of advanced-stage serous ovarian cancers distinguishes novel subclasses and implicates zeb2 in tumor progression and prognosis. Cancer Sci..

[100] Sun B., Nishihira J., Suzuki M., Fukushima N., Ishibashi T., Kondo M., Sato Y., Todo S. (2003). Induction of macrophage migration inhibitory factor by lysophosphatidic acid: Relevance to tumor growth and angiogenesis. Int. J. Mol. Med..

[101] Fang X., Schummer M., Mao M., Yu S., Tabassam F.H., Swaby R., Hasegawa Y., Tanyi J.L., LaPushin R., Eder A. (2002). Lysophosphatidic acid is a bioactive mediator in ovarian cancer. Biochim. Biophys. Acta.

[102] Baker D.L., Morrison P., Miller B., Riely C.A., Tolley B., Westermann A.M., Bonfrer J.M., Bais E., Moolenaar W.H., Tigyi G. (2002). Plasma lysophosphatidic acid concentration and ovarian cancer. JAMA.

[103] Mao X.Y., Lee M.J., Zhu J., Zhu C., Law S.M., Snijders A.M. (2017). Genome-wide screen identifies a novel prognostic signature for breast cancer survival. Oncotarget.

[104] Kok B.P., Venkatraman G., Capatos D., Brindley D.N. (2012). Unlike two peas in a pod: Lipid phosphate phosphatases and phosphatidate phosphatases. Chem. Rev..

[105] Morris A.J., Smyth S.S. (2014). Lipid phosphate phosphatases: More than one way to put the brakes on lpa signaling?. J. Lipid Res..

[106] Tang X., Benesch M.G., Dewald J., Zhao Y.Y., Patwardhan N., Santos W.L., Curtis J.M., McMullen T.P., Brindley D.N. (2014). Lipid phosphate phosphatase-1 expression in cancer cells attenuates tumor growth and metastasis in mice. J. Lipid Res..

[107] Pilquil C., Dewald J., Cherney A., Gorshkova I., Tigyi G., English D., Natarajan V., Brindley D.N. (2006). Lipid phosphate phosphatase-1 regulates lysophosphatidate-induced fibroblast migration by controlling phospholipase d2-dependent phosphatidate generation. J. Biol. Chem..

[108] Alderton F., Darroch P., Sambi B., McKie A., Ahmed I.S., Pyne N., Pyne S. (2001). G-protein-coupled receptor stimulation of the p42/p44 mitogen-activated protein kinase pathway is attenuated by lipid phosphate phosphatases 1, 1a, and 2 in human embryonic kidney 293 cells. J. Biol. Chem..

[109] Nakayama J., Raines T.A., Lynch K.R., Slack-Davis J.K. (2015). Decreased peritoneal ovarian cancer growth in mice lacking expression of lipid phosphate phosphohydrolase 1. PLoS ONE.

[110] Flanagan J.M., Funes J.M., Henderson S., Wild L., Carey N., Boshoff C. (2009). Genomics screen in transformed stem cells reveals RNASEH2A, PPAP2C, and ADARB1 as putative anticancer drug targets. Mol. Cancer Ther..

[111] Morris K.E., Schang L.M., Brindley D.N. (2006). Lipid phosphate phosphatase-2 activity regulates s-phase entry of the cell cycle in rat2 fibroblasts. J. Biol. Chem..

[112] Clarke N., Arenzana N., Hai T., Minden A., Prywes R. (1998). Epidermal growth factor induction of the c-jun promoter by a rac pathway. Mol. Cell. Biol..

[113] Crowe D.L., Tsang K.J., Shemirani B. (2001). Jun n-terminal kinase 1 mediates transcriptional induction of matrix metalloproteinase 9 expression. Neoplasia.

[114] Balogh A., Shimizu Y., Lee S.C., Norman D.D., Gangwar R., Bavaria M., Moon C., Shukla P., Rao R., Ray R. (2015). The autotaxin-LPA2 GPCR axis is modulated by gamma-irradiation and facilitates DNA damage repair. Cell Signal..

[115] Venkatraman G., Benesch M.G., Tang X., Dewald J., McMullen T.P., Brindley D.N. (2015). Lysophosphatidate signaling stabilizes Nrf2 and increases the expression of genes involved in drug resistance and oxidative stress responses: Implications for cancer treatment. FASEB J..

[116] Smith B.D., Bellon J.R., Blitzblau R., Freedman G., Haffty B., Hahn C., Halberg F., Hoffman K., Horst K., Moran J. (2018). Radiation therapy for the whole breast: Executive summary of an american society for radiation oncology (astro) evidence-based guideline. Pract. Radiat. Oncol..

[117] Meng G., Tang X., Yang Z., Benesch M.G.K., Marshall A., Murray D., Hemmings D.G., Wuest F., McMullen T.P.W., Brindley D.N. (2017). Implications for breast cancer treatment from increased autotaxin production in adipose tissue after radiotherapy. FASEB J..

[118] Meng G., Wuest M., Tang X., Dufour J., Zhao Y., Curtis J.M., McMullen T.P.W., Murray D., Wuest F., Brindley D.N. (2019). Repeated fractions of x-radiation to the breast fat pads of mice augment activation of the autotaxin-lysophosphatidate-inflammatory cycle. Cancers.

[119] Jayakumar S., Pal D., Sandur S.K. (2015). Nrf2 facilitates repair of radiation induced DNA damage through homologous recombination repair pathway in a ros independent manner in cancer cells. Mutat. Res..

[120] Sekhar K.R., Freeman M.L. (2015). Nrf2 promotes survival following exposure to ionizing radiation. Free Radic. Biol. Med..

[121] Zhou S., Ye W., Shao Q., Zhang M., Liang J. (2013). Nrf2 is a potential therapeutic target in radioresistance in human cancer. Crit. Rev. Oncol. Hematol..

[122] Dalamaga M., Diakopoulos K.N., Mantzoros C.S. (2012). The role of adiponectin in cancer: A review of current evidence. Endocr. Rev..

[123] Deng W., Balazs L., Wang D.A., Van Middlesworth L., Tigyi G., Johnson L.R. (2002). Lysophosphatidic acid protects and rescues intestinal epithelial cells from radiation- and chemotherapy-induced apoptosis. Gastroenterology.

[124] Deng W., Shuyu E., Tsukahara R., Valentine W.J., Durgam G., Gududuru V., Balazs L., Manickam V., Arsura M., VanMiddlesworth L. (2007). The lysophosphatidic acid type 2 receptor is required for protection against radiation-induced intestinal injury. Gastroenterology.

[125] Brown J.M., Attardi L.D. (2005). The role of apoptosis in cancer development and treatment response. Nat. Rev. Cancer.

[126] Tang X., Wuest M., Benesch M.G.K., Dufour J., Zhao Y., Curtis J.M., Monjardet A., Heckmann B., Murray D., Wuest F. (2020). Inhibition of autotaxin with GLPG1690 increases the efficacy of radiotherapy and chemotherapy in a mouse model of breast cancer. Mol. Cancer Ther..

[127] Brindley D.N., Rolland Y. (1989). Possible connections between stress, diabetes, obesity, hypertension and altered lipoprotein metabolism that may result in atherosclerosis. Clin. Sci..

[128] Brindley D.N., Wang C.-N., O’Brien L., Mei J. (1998). Insulin resistance: The role of glucocorticoids, fatty acids and tumor necrosis factor-a. Can. J. Diabetes Care.

[129] Cao P., Aoki Y., Badri L., Walker N.M., Manning C.M., Lagstein A., Fearon E.R., Lama V.N. (2017). Autocrine lysophosphatidic acid signaling activates beta-catenin and promotes lung allograft fibrosis. J. Clin. Investig..

[130] Erstad D.J., Tager A.M., Hoshida Y., Fuchs B.C. (2017). The autotaxin-lysophosphatidic acid pathway emerges as a therapeutic target to prevent liver cancer. Mol. Cell Oncol..

[131] Farquhar M.J., Humphreys I.S., Rudge S.A., Wilson G.K., Bhattacharya B., Ciaccia M., Hu K., Zhang Q., Mailly L., Reynolds G.M. (2017). Autotaxin-lysophosphatidic acid receptor signalling regulates hepatitis c virus replication. J. Hepatol..

[132] Gan L., Xue J.X., Li X., Liu D.S., Ge Y., Ni P.Y., Deng L., Lu Y., Jiang W. (2011). Blockade of lysophosphatidic acid receptors LPAR1/3 ameliorates lung fibrosis induced by irradiation. Biochem. Biophys. Res. Commun..

[133] Kaffe E., Katsifa A., Xylourgidis N., Ninou I., Zannikou M., Harokopos V., Foka P., Dimitriadis A., Evangelou K., Moulas A.N. (2017). Hepatocyte autotaxin expression promotes liver fibrosis and cancer. Hepatology.

[134] Oikonomou N., Mouratis M.A., Tzouvelekis A., Kaffe E., Valavanis C., Vilaras G., Karameris A., Prestwich G.D., Bouros D., Aidinis V. (2012). Pulmonary autotaxin expression contributes to the pathogenesis of pulmonary fibrosis. Am. J. Respir. Cell Mol. Biol..

[135] Okudaira S., Yukiura H., Aoki J. (2010). Biological roles of lysophosphatidic acid signaling through its production by autotaxin. Biochimie.

[136] Pradere J.P., Klein J., Gres S., Guigne C., Neau E., Valet P., Calise D., Chun J., Bascands J.L., Saulnier-Blache J.S. (2007). LPA1 receptor activation promotes renal interstitial fibrosis. J. Am. Soc. Nephrol..

[137] Rancoule C., Pradere J.P., Gonzalez J., Klein J., Valet P., Bascands J.L., Schanstra J.P., Saulnier-Blache J.S. (2011). Lysophosphatidic acid-1-receptor targeting agents for fibrosis. Expert Opin. Investig. Drugs.

[138] Sevastou I., Kaffe E., Mouratis M.A., Aidinis V. (2013). Lysoglycerophospholipids in chronic inflammatory disorders: The PLA(2)/LPC and ATX/LPA axes. Biochim. Biophys. Acta.

[139] Swaney J.S., Chapman C., Correa L.D., Stebbins K.J., Bundey R.A., Prodanovich P.C., Fagan P., Baccei C.S., Santini A.M., Hutchinson J.H. (2010). A novel, orally active lpa(1) receptor antagonist inhibits lung fibrosis in the mouse bleomycin model. Br. J. Pharmacol..

[140] Zhao Y., Natarajan V. (2013). Lysophosphatidic acid (lpa) and its receptors: Role in airway inflammation and remodeling. Biochim. Biophys. Acta.

[141] Tager A.M., LaCamera P., Shea B.S., Campanella G.S., Selman M., Zhao Z., Polosukhin V., Wain J., Karimi-Shah B.A., Kim N.D. (2008). The lysophosphatidic acid receptor lpa1 links pulmonary fibrosis to lung injury by mediating fibroblast recruitment and vascular leak. Nat. Med..

[142] Nikolaou A., Ninou I., Kokotou M.G., Kaffe E., Afantitis A., Aidinis V., Kokotos G. (2018). Hydroxamic acids constitute a novel class of autotaxin inhibitors that exhibit in vivo efficacy in a pulmonary fibrosis model. J. Med. Chem..

[143] Povirk L.F. (1996). DNA damage and mutagenesis by radiomimetic DNA-cleaving agents: Bleomycin, neocarzinostatin and other enediynes. Mutat. Res..

[144] Maher T.M., Kreuter M., Lederer D.J., Brown K.K., Wuyts W., Verbruggen N., Stutvoet S., Fieuw A., Ford P., Abi-Saab W. (2019). Rationale, design and objectives of two phase iii, randomised, placebo-controlled studies of GLPG1690, a novel autotaxin inhibitor, in idiopathic pulmonary fibrosis (ISABELA 1 and 2). BMJ Open Respir. Res..

[145] Palmer S.M., Snyder L., Todd J.L., Soule B., Christian R., Anstrom K., Luo Y., Gagnon R., Rosen G. (2018). Randomized, double-blind, placebo-controlled, phase 2 trial of bms-986020, a lysophosphatidic acid receptor antagonist for the treatment of idiopathic pulmonary fibrosis. Chest.

[146] Meng G., Wuest M., Tang X., Dufour J., McMullen T.P.W., Wuest F., Murray D., Brindley D.N. (2020). Dexamethasone attenuates x-ray-induced activation of the autotaxin-lysophosphatidate-inflammatory cycle in breast tissue and subsequent breast fibrosis. Cancers.

[147] Van Meeteren L.A., Moolenaar W.H. (2007). Regulation and biological activities of the autotaxin-lpa axis. Prog. Lipid Res..

[148] Shea B.S., Tager A.M. (2012). Role of the lysophospholipid mediators lysophosphatidic acid and sphingosine 1-phosphate in lung fibrosis. Proc. Am. Thorac. Soc..

[149] Miyabe C., Miyabe Y., Nagai J., Miura N.N., Ohno N., Chun J., Tsuboi R., Ueda H., Miyasaka M., Miyasaka N. (2019). Abrogation of lysophosphatidic acid receptor 1 ameliorates murine vasculitis. Arthritis Res. Ther..

[150] Funke M., Zhao Z., Xu Y., Chun J., Tager A.M. (2012). The lysophosphatidic acid receptor lpa1 promotes epithelial cell apoptosis after lung injury. Am. J. Respir. Cell Mol. Biol..

[151] Nincheri P., Bernacchioni C., Cencetti F., Donati C., Bruni P. (2010). Sphingosine kinase-1/S1P1 signalling axis negatively regulates mitogenic response elicited by PDGF in mouse myoblasts. Cell Signal..

[152] Tager A.M. (2012). Autotaxin emerges as a therapeutic target for idiopathic pulmonary fibrosis: Limiting fibrosis by limiting lysophosphatidic acid synthesis. Am. J. Respir. Cell Mol. Biol..

[153] Hong J.H., Chiang C.S., Tsao C.Y., Lin P.Y., McBride W.H., Wu C.J. (1999). Rapid induction of cytokine gene expression in the lung after single and fractionated doses of radiation. Int. J. Radiat. Biol..

[154] Hong J.H., Chiang C.S., Tsao C.Y., Lin P.Y., Wu C.J., McBride W.H. (2001). Can short-term administration of dexamethasone abrogate radiation-induced acute cytokine gene response in lung and modify subsequent molecular responses?. Int. J. Radiat. Oncol. Biol. Phys..

[155] Wang L.P., Wang Y.W., Wang B.Z., Sun G.M., Wang X.Y., Xu J.L. (2014). Expression of interleukin-17a in lung tissues of irradiated mice and the influence of dexamethasone. Sci. World J..

[156] Liu Z.H., Fan W., Chen R.C. (2015). 3,4-dihydroxyphenylethanol suppresses irradiation-induced pulmonary fibrosis in adult rats. Int. J. Clin. Exp. Pathol..

[157] Ward H.E., Kemsley L., Davies L., Holecek M., Berend N. (1993). The effect of steroids on radiation-induced lung disease in the rat. Radiat. Res..

[158] Allanore Y., Distler O., Jagerschmidt A., Illiano S., Ledein L., Boitier E., Agueusop I., Denton C.P., Khanna D. (2018). Lysophosphatidic acid receptor 1 antagonist sar100842 for patients with diffuse cutaneous systemic sclerosis: A double-blind, randomized, eight-week placebo-controlled study followed by a sixteen-week open-label extension study. Arthritis Rheumatol..

